# Upregulation of TLR4 via PKC activation contributes to impaired wound healing in high-glucose-treated kidney proximal tubular cells

**DOI:** 10.1371/journal.pone.0178147

**Published:** 2017-05-24

**Authors:** Jianping Peng, Hang Zheng, Xia Wang, Zhixiang Cheng

**Affiliations:** 1 Department of Urology, Zhongnan Hospital of Wuhan University, Wuhan University, Wuhan, China; 2 Department of Internal Medicine, Hospital of Wuhan University, Wuhan University, Wuhan, China; 3 Department of General Surgery, Zhongnan Hospital of Wuhan University, Wuhan University, Wuhan, China; Universitatsklinikum Hamburg-Eppendorf, GERMANY

## Abstract

Acute kidney injury (AKI) leads to a worse prognosis in diabetic patients compared with prognoses in non-diabetic patients, but whether and how diabetes affects kidney repair after AKI remains unknown. Here, we used scratch-wound healing and transwell migration models to examine whether and how wound healing is affected by high glucose levels in cultured kidney proximal tubular cells (RPTC). The results show that scratch-wound healing and transwell migration were significantly slower in high-glucose-treated kidney tubular cells (30 mM glucose) than in low-glucose-treated cells (5.5 mM). Toll-like receptor 4 (TLR4), MyD88, phospho-protein kinase C (PKC), phospho-p38 MAPK and monocyte chemoattractant protein-1 (MCP-1) mRNA levels were upregulated after high glucose treatments. Staurosporine, a selective PKC inhibitor, inhibited TLR4, MyD88 and p-p38 upregulation in the high-glucose-treated cells, indicating the involvement of PKC in high-glucose-induced TLR4 upregulation. The pharmacological inhibition of TLR4 or shRNA-mediated TLR4 knockdown improved wound healing and transwell migration in high-glucose-treated RPTC. In contrast, the overexpression of TLR4 in low-glucose-treated RPTC suppressed wound healing, mimicking the effects of high glucose levels. These results suggest that the upregulation of TLR4 expression via PKC activation contributes to defective wound healing in high-glucose-treated kidney tubular cells.

## Introduction

Acute kidney injury (AKI) leads to significantly lower recovery rates and higher mortality rates in diabetic patients compared with rates in non-diabetic patients[1]. Recent research has gained significant insight into the mechanism underlying these trends. Goor *et al*. demonstrated the greater vulnerability of STZ-induced diabetic rats to ischemic AKI[2], and our recent study demonstrates that renal ischemia-reperfusion induces more severe AKI in diabetic mice than in nondiabetic mice and that the severity of AKI in these mice is correlated with their blood glucose levels[3]. However, while the pathophysiology of AKI includes kidney tubular cell death and repair[4], most studies have just focused on tubule death, and kidney repair after AKI has received much less attention. Whether and how kidney repair after AKI is affected by high glucose levels has not been clarified.

Toll-like receptors (TLRs) modulate immune responses and inflammatory diseases, and the activation of TLR4 through the adapter molecules MyD88 leads to the transcriptional expression of proinflammatory cytokines and chemokines, such as NF-κB p38 and monocyte chemoattractant protein-1 (MCP-1), resulting in the upregulation of distinct target genes[5]. The activation of TLR4 modifies tissue injury and repair in both positive or negative ways. TLR4 promotes injury in almost all organs as demonstrated by the protection observed in TLR4-mutant or TLR4-deficient mice after hepatic, renal, cardiac, and cerebral ischemia-reperfusion[6–8], but it exerts a cytoprotective role and prevents tissue injury under stressful conditions in the lungs and intestine[9, 10]. In skin wounds, TLR4 is upregulated in epidermal keratinocytes during the injury and healing phases[11, 12], and TLR4-deficient mice show impaired wound healing in their skin[11], indicating that TLR4 promotes wound healing in skin. However, TLR4 plays a negative role under diabetic conditions, as indicated by the fact that skin wound healing is significantly improved in TLR4-deficient mice with induced diabetes compared to healing in diabetic wild-type animals[13]. This suggests that TLR4 inhibits skin wound healing in the presence of high glucose levels and that TLR4 contributes to impaired skin wound healing in the presence of high glucose levels. In kidneys, TLR4 and TLR2 promote injury in response to renal ischemia-reperfusion[6, 14], TLR4 is activated in the renal tubules of human kidneys with diabetic nephropathy, where it promotes tubular inflammation[15]; however, the precise role of TLR4 in kidney tubular cell repair under high glucose conditions remains unknown.

In this study, we first demonstrated the inhibitory effect of high glucose levels on wound healing in kidney tubular cells. Then, we examined the role of TLR4 in the impaired wound healing observed under high glucose conditions. The results suggest that TLR4 contributes to the wound healing defects observed in kidney proximal tubular cells at high glucose levels *in vitro*.

## Materials and methods

### Antibodies and special reagents

Antibodies were purchased from the following sources: polyclonal anti-TLR4, anti-PKC-pan (phospho-Thr497), total PKC, phosphor-p38 MAPK, MyD88 were from Cell Signaling Technology (Danvers, MA), the secondary antibodies used for the immunoblot analysis were from Jackson ImmunoResearch Laboratories Inc. (West Grove, PA), DMOG and staurosporine were from Sigma (St. Louis, MO), and TAK-242 was from MedChem Express (Monmouth Junction, NJ).

### High-glucose cell treatment

High-glucose treatments were conducted as described in our recent studies[3]. Immortalized rat kidney proximal tubular cells (RPTC) were originally obtained from Dr. Ulrich Hopfer (Case Western Reserve University, Cleveland, OH). NRK-52E and HEK 293 cells were purchased from the Cell Resource Center of Shanghai Institutes. For the high-glucose treatment, tubular cells were cultured for 2 days in a medium containing 30 mmol/L glucose, a concentration previously used for *in vitro* hyperglycemic treatments[16]. The control cells were grown in media containing 5.5 mmol/L glucose (100 mg/dL, normal glucose) or in 5.5 mmol/L glucose with 24.5 mmol/L mannitol.

### Scratch-wound healing assay

Scratch-wound healing assay was conducted as described in our recent studies[17]. Cell proliferation was not blocked with mitomycin or other substance. For morphological examinations, a scratch-wound healing model was used. In brief, a monolayer of confluent RPTC grown in a 35-mm dish was linearly scratched with a sterile 1000 μL pipette tip. Phase-contrast images were recorded at 0 and 18 h after scratching. The width of the wound was measured at various time points to determine the distance over which healing occurred. Whole-cell lysates were collected at different time points after wounding for immunoblot or PCR analyses.

### Transwell cell migration assay

Transwell cell migration was measured as described previously[3]. Cell proliferation was not blocked with mitomycin or other substance, because the time-points images showed that there was no obvious proliferation at first 6 hours during scratch wound healing (S1 Fig). In brief, the undersurfaces of transwells (Costar; Corning Life Sciences, Lowell, MA) were coated with 10 μg/mL collagen I (Millipore) overnight at 4°C. Coated wells were then placed into a 24-well plate containing 600 μL of culture medium. RPTC were detached and suspended at 1.5 x 10^6^ cells/mL in culture medium. The cells were then added into the transwells (200 μL, 3 x 10^5^ cells/well) and allowed to migrate for 6 h at 37°C. Cotton swabs were used to remove cells from the upper surface of the transwells, and migratory cells attached to the undersurface were stained with propidium iodide (PI). The numbers of migrated cells were counted using an inverted microscope. To determine the effect of TLR4 inhibitors on cell migration, the inhibitors were added to the medium in the bottom chamber.

### Lentiviral shRNA-mediated TLR4 knockdown and TLR4 overexpression

Lentiviral shRNA plasmids targeting TLR4 and a scrambled non-targeting control plasmid were made using the pLV-mU6-EF1a-GFP vector (Biosettia, San Diego, CA). The target sequence of the TLR4 shRNA was GCATAGAGGTACTTCCTAATA. Rat TLR4 cDNA was used as the template for PCR-based deletion to generate TLR4, which was subcloned into the lentiviral vector pCDH-CMV-MCS-EF1-copGFP (System Biosciences, Mountain View, CA). These TLR4 plasmids were cotransfected into 293FT cells (Invitrogen, Carlsbad, CA) with three packaging plasmids (pLP1, pLP2, and pLP/VSV-G), and the culture medium was collected at 48 h. The culture medium with the packaged lentiviruses was added to RPTC for 1 day for infection. The medium was then replaced with fresh medium for an additional 2 days of culture before the wound-healing test.

### Real-time PCR

Total RNA was extracted using the RNeasy Mini Kit (Applied Biosystems Ambion, Austin, TX) and was reverse transcribed to cDNA with M-MLV reverse transcriptase (Applied Biosystems). TLR4 and monocyte chemoattractant protein-1 (MCP-1) gene expression was analyzed via real-time PCR. Quantification was performed using the ΔCt values.

### Immunoblot analysis

Protein concentration was determined using a BCA reagent (Thermo Fisher Scientific, Waltham, MA). Equal amounts of protein were loaded into each well of gels for electrophoresis using the NuPAGE Gel System (Invitrogen). Proteins were then transferred onto polyvinylidene fluoride membranes. The blots were then incubated in blocking buffer for 1 h and then with primary antibodies overnight at 4°C. After being washed, the blots were incubated with a horseradish peroxidase-conjugated secondary antibody, and the antigens on the blots were revealed using the enhanced chemiluminescence kit from Thermo Fisher Scientific.

### Immunofluorescence

RPTC were grown on glass coverslips and cultured in low glucose medium or high glucose medium for 2 days, Then the cells were fixed with 4% paraformaldehyde and permeabilized in a blocking buffer. The cells were then exposed to TLR4 antibody, then incubated with Cy3-labeled secondary antibody, and examined by fluorescence microscopy using the Cy3 channel.

### Statistical analysis

Quantitative data are expressed as the means ± S.D. Significant differences between two groups were determined using Student’s t-tests. Results with P<0.05 were considered significantly different. Qualitative results, including those from immunoblots, are representative of at least three separate experiments.

## Results

### High glucose inhibits scratch-wound healing in cultured kidney tubular cells

**Fig 1:**
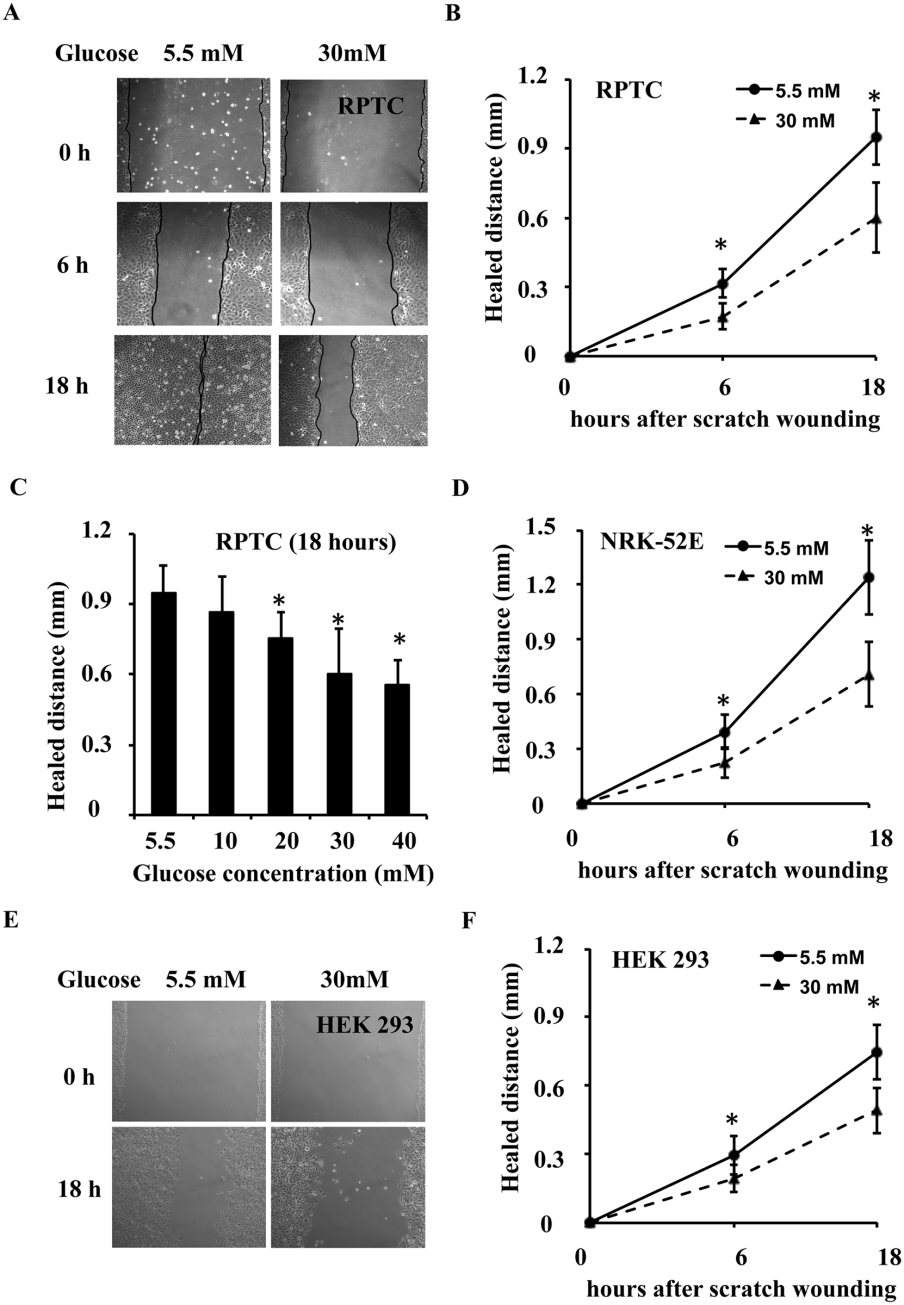
High glucose levels inhibit scratch-wound healing in cultured kidney tubular cells. RPTC, NRK-52E and HEK 293 cells were cultured for 2 days in low glucose (5.5 mM) or high glucose (30 mM) medium, followed by a scratch-wound healing experiment. (A) RPTC were scratch-wounded with a sterile pipette tip and then incubated in low glucose or high glucose medium. Representative wounds immediately after scratching and after 6 and 18 h of healing were recorded with a phase-contrast microscope. (B) The wound width was measured at 6 and 18 h after scratching to determine the distance over which healing occurred in RPTC. (C) High glucose inhibited scratch-wound healing in RPTC in a concentration-dependent manner at 18 h after scratching. (D) NRK-52E were scratch-wounded with a sterile pipette tip and then incubated in low glucose or high glucose medium, the wound width was measured at 18 h after scratching. (E and F) HEK 293 were scratch-wounded with a sterile pipette tip and then incubated in low glucose or high glucose medium, the wound width was measured at 18 h after scratching. Data are expressed as the mean ± S.D. (n = 4). *, p<0.05 versus the low glucose group.

Whether and how wound healing is affected by high glucose after AKI is largely unknown. We examined the effect of high glucose levels in a scratch-wound healing model of cultured kidney tubular cells, including RPTC, NRK-52E and HEK 293 cells. In this model, tubular cells were cultured in low glucose (5.5 mM) and high glucose (30 mM) medium for 2 days. These cell cultures were then scratch-wounded with a sterile pipette tip. Wounds were recorded and measured immediately after scratching (0 h) and at different time points post-scratching. Wounds in RPTC are shown in Fig 1A. In low glucose medium, most of the wounds healed within 18 h. However, a significant wound remained in RPTC in the high glucose medium. Measurements of the distance over which healing occurred show that wound healing was suppressed at high glucose levels in a time-dependent manner (Fig 1B). High glucose inhibited scratch-wound healing in RPTC in a concentration-dependent manner (Fig 1C). Similar results were seen in NRK-52E cells (Fig 1D) and HEK 293 cells (Fig 1E and 1F).

### High glucose inhibits transwell migration in cultured kidney tubular cells

**Fig 2:**
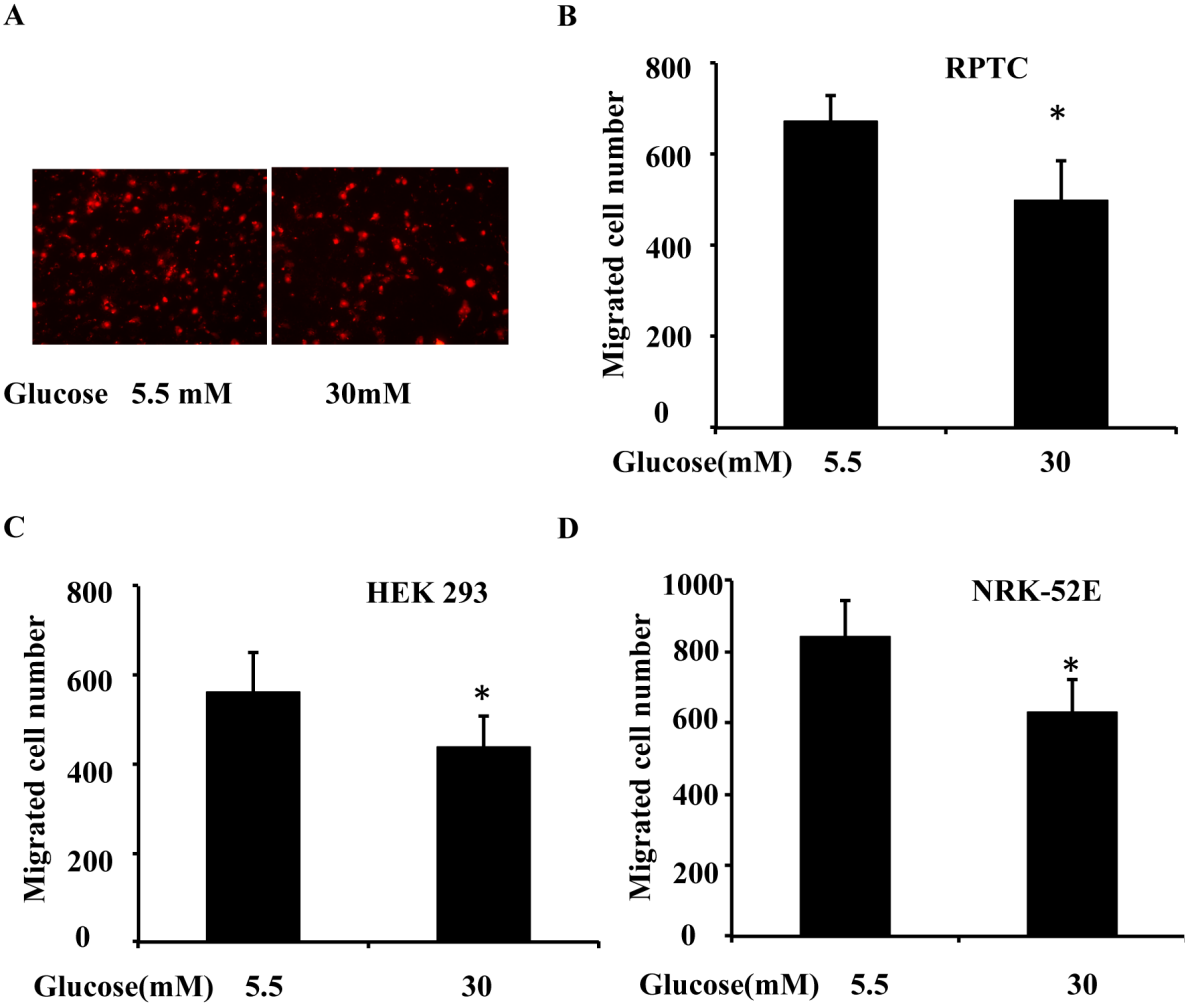
High glucose inhibits transwell migration in cultured kidney tubular cells. RPTC, NRK-52E and HEK 293 cells were cultured for 2 days in low glucose (5.5 mM) or high glucose (30 mM) medium, and then used for a transwell migration experiment. (A) Representative PI staining of migratory cells was recorded with a fluorescence microscope. A total of 3x10^5^ RPTC were seeded in a transwell insert, which was put in a 24-well plate containing 600 μL low glucose or high glucose medium for 6 h. The cells that migrated to the undersurface were stained with PI and counted. (B) Migratory cells attached to the undersurface were counted after PI staining in RPTC. (C) Migratory cells attached to the undersurface were counted after PI staining in HEK 293 cells. (D) Migratory cells attached to the undersurface were counted after PI staining in NRK-52E. Data are expressed as the mean ± S.D. (n = 4). *, p<0.05 versus the low glucose group.

Wound healing involves a rapid mobilization of the cells at the edge of the wound and migration into the wound in the scratch model. We hypothesized that high glucose levels might inhibit wound healing in part by blocking cell migration. To test this possibility, we determined the effect of high glucose levels on cell migration using a transwell cell migration assay in RPTC, NRK-52E and HEK 293 cells. As shown in Fig 2A, fewer RPTC migrated in the high glucose condition than in the low glucose condition. Cell counting showed that in 6 h, approximately 670 cells migrated in the low glucose medium, whereas approximately 490 did so in the high glucose medium (Fig 2B). Similar results were seen in HEK 293 cells (Fig 2C) and NRK-52E cells (Fig 2D)

### TLR4 upregulation during high glucose treatment in RPTC

**Fig 3:**
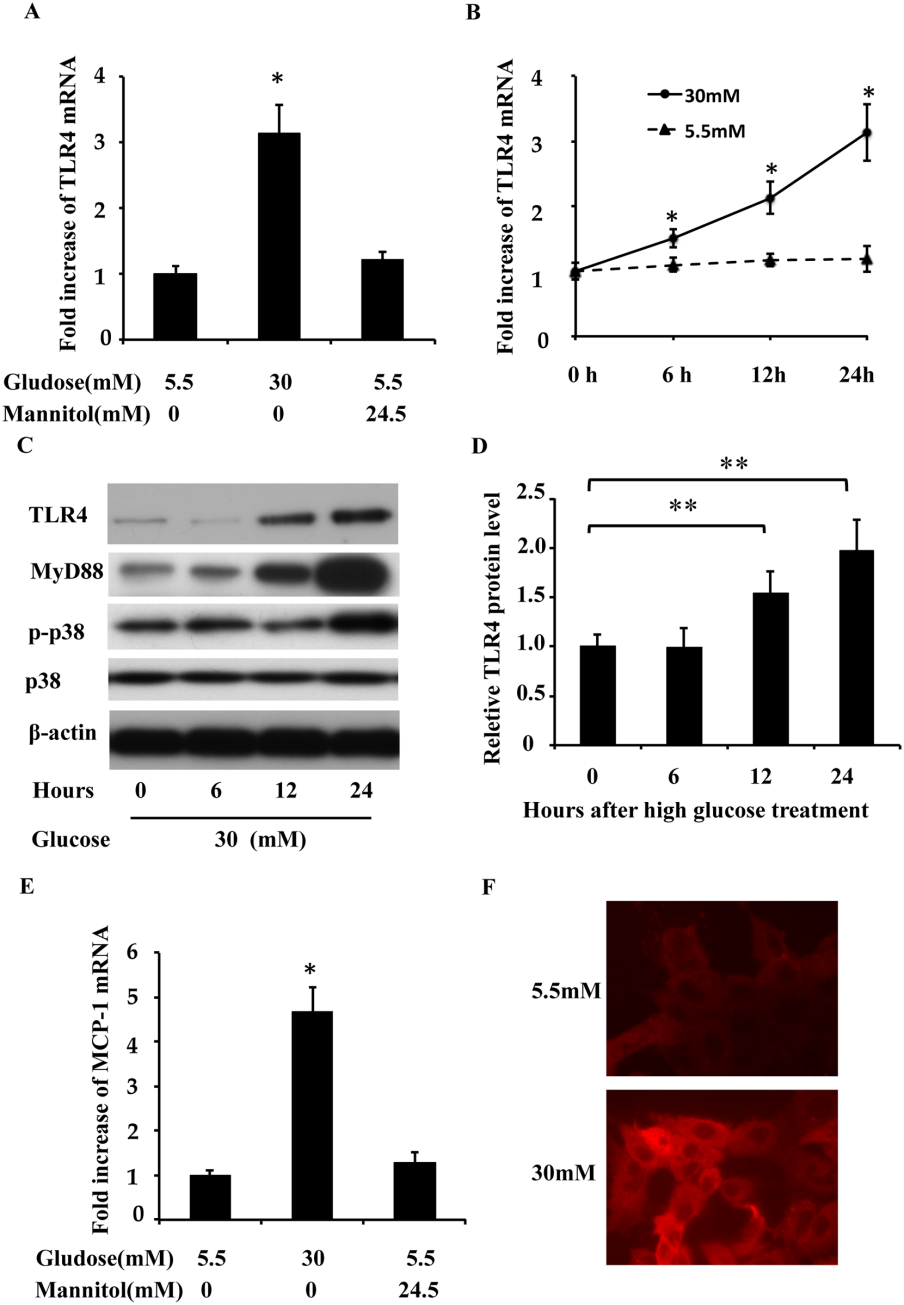
TLR4 upregulation during high glucose treatment in RPTC. TLR4 mRNA and protein levels were examined via real-time PCR and Western blotting after low gucose or high glucose treatment. (A and B) TLR4 mRNA upregulation in the high glucose medium. (C) TLR4 protein levels increased after the high glucose treatment. (D) Experiments were analyzed via densitometry and normalized to the control (0 h). (E) MCP-1 mRNA upregulation in high glucose medium for 24 h. (F) Immunofluorescence analysis of TLR4 expression. PRTC cells were cultured for 2 days in low glucose (5.5 mM) or high glucose (30 mM) medium.The cells were fixed for immunofluorescent staining of TLR4 (red). Data are expressed as the mean ± S.D. (n≥3). *, p<0.05 versus the low glucose group; **, p<0.05 versus the control (0 h).

To understand the mechanism of wound healing defects in high-glucose-treated cells, we examined the expression of TLR4, MyD88, p-p38, monocyte chemoattractant protein-1 (MCP-1) protein levels and TLR4 mRNA. RPTC were cultured in low glucose and high glucose medium for 24 hours, and the cell lysates were collected at different time points. TLR4 mRNA and protein levels were examined via real-time PCR and Western blotting. As shown in Fig 3A and 3B, high glucose levels induced TLR4 mRNA expression in a time-dependent manner, with a peak stimulation of 3.1-fold after 24 hours of exposure, whereas exposure to an equivalent dose of mannitol had no effect (Fig 3A). TLR4 and MyD88 protein upregulation was detectable at 12 h after high glucose treatment and was further increased at 24 h, while as p-p38 level increased at 24 h (Fig 3C), but upregulation not detected in low-glucose-treated RPTC. The time-dependent changes in TLR4 expression after high glucose treatment were further confirmed through quantification via densitometry (Fig 3D). The mRNA expression of MCP-1, which is a downstream signaling target of TLR4 activation, was significantly higher in high-glucose-treated RPTC (Fig 3E). We further examined TLR4 expression by immunofluorescence (Fig 3F), TLR4 was detected in the cytoplasm of the majority of RPTC cells and seemed to be more intense under high glucose conditions.

### PKC is involved in high-glucose-induced TLR4 upregulation in RPTC

**Fig 4:**
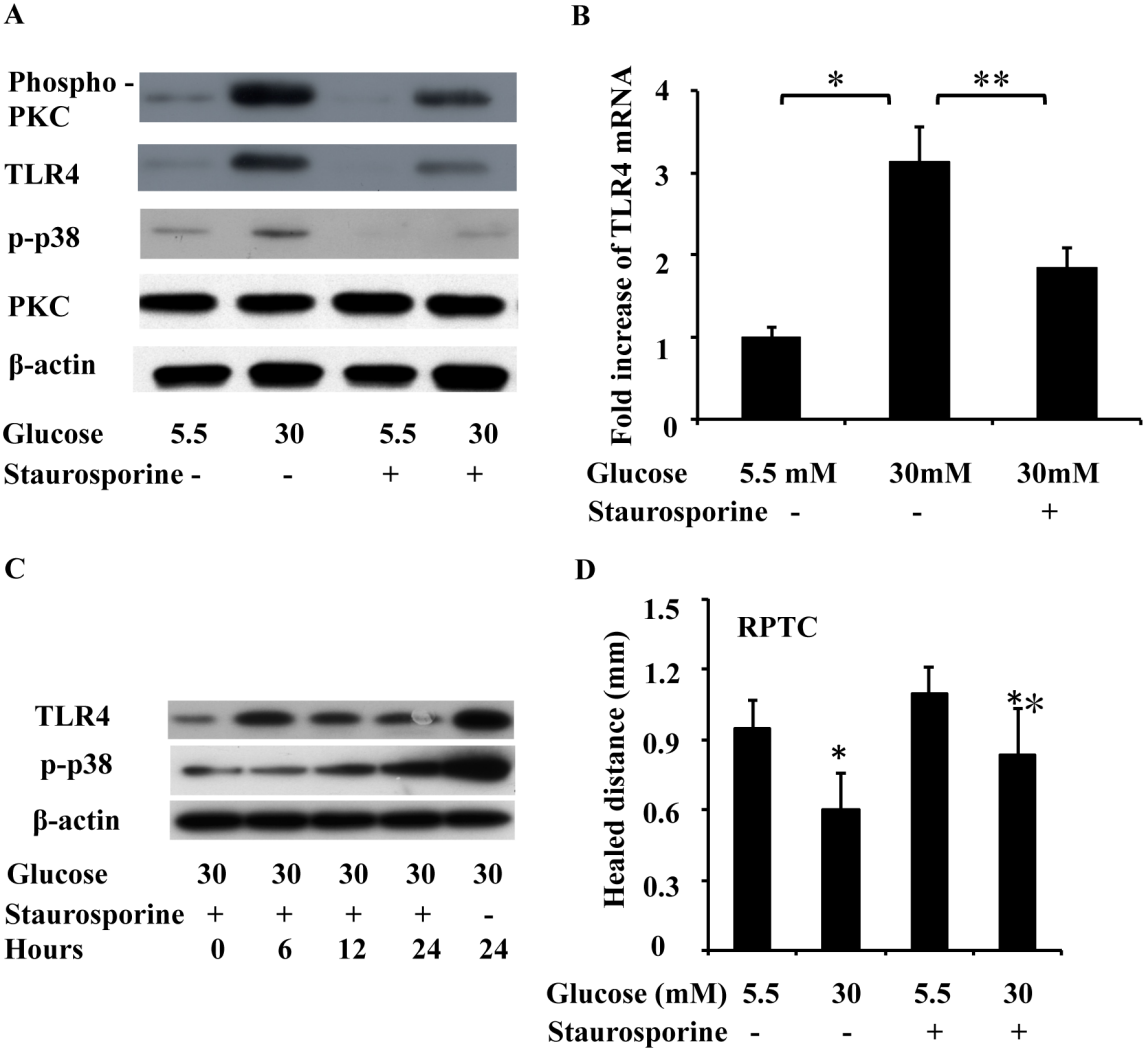
Effect of PKC on high glucose induced TLR4 expression. RPTC were treated for 24 hours with low glucose (5.5 mM), high glucose (30 mM), or the PKC inhibitor staurosporine (10 nM, added 1 hour before high glucose treatment), and the cell lysates were collected, TLR4 mRNA, phospho-PKC (pan), p-p38, p38 levels were examined via real-time PCR and Western blotting. (A) PKC phosphorylation and p-p38 upregulation after high glucose treatment. The PKC inhibitor staurosporine decreased phospho-PKC and p-p38 levels but not total PKC levels. (B) Staurosporine reduced TLR4 mRNA levels under the high glucose condition. (C) RPTC were pretreated with staurosporine (10 nM), then treated with high glucose (30mM) for 6, 12, 24 h, and cell lysates were collected at different time points. phospho-PKC (pan), p-p38 levels were examined via Western blotting. (D) Effect of staurosporine on scratch wound healing in low glucose and high glucose. Data are expressed as the mean ± S.D. (n = 4). *, p<0.05 versus the low glucose group; **, p<0.05 versus the high glucose group without staurosporine treatment.

To investigate the mechanism of high-glucose-induced TLR4 expression, we next examined the role of PKC in the effects of high glucose levels. PKC-pan (phospho-Thr497) and phospho-p38 (p-p38) were increased at 24 h after high glucose treatment, while total PKC levels showed no significant change (Fig 4A). Pretreatment of RPTC with staurosporine (10 nM), which is a PKC inhibitor, 1 hour before high glucose exposure decreased the phospho-PKC level and resulted in a 41% decrease in the subsequent TLR4 mRNA overexpression (Fig 4B), and staurosporine also blocked the upregulation of phospho-PKC and p-p38 induced by high glucose (Fig 4A), indicating the involvement of PKC in TLR4 upregulation in response to high glucose levels. We next examined the effects of this PKC inhibitor on scratch-wound healing, as shown in Fig 4D, staurosporine significantly promoted wound healing under high glucose conditions. Further time-course experiment revealed that staurosporine blocked TLR4 upregulation at 6 h after high glucose treatment, but inhibited p-p38 induction at 12 h after high glucose treatment (Fig 4C).

### TLR4 inhibitor promotes wound healing in high glucose conditions

**Fig 5:**
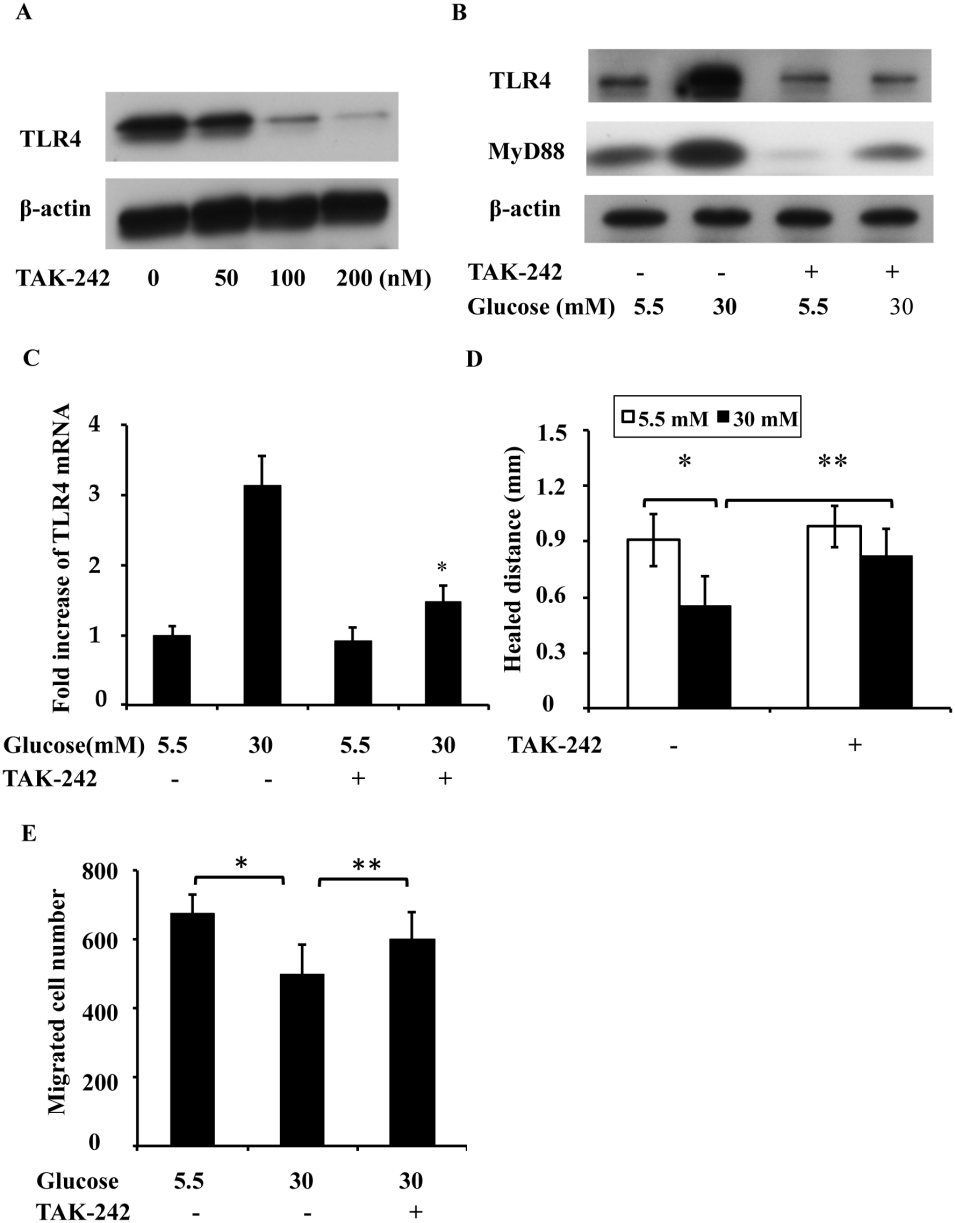
TLR4 inhibitor promotes scratch-wound healing and migration in high-glucose-treated RPTC. RPTC were cultured for 2 days in low glucose (5.5 mM) or high glucose (30 mM) medium, and then used for following experiments. (A) Titration of TAK-242 concentration. RPTC cells were cultured for 12 h in low glucose or high glucose medium with or without TAK-242. (B) MyD88 upregulation was also inhibited by TAK-242 under high glucose conditions by Western blotting. (C) TLR4 mRNA upregulation was also inhibited by TAK-242 under high glucose conditions by real-time PCR analysis. (D) Enhanced wound healing in the high glucose condition with the TLR4 inhibitor. RPTC cells were scratch-wounded and incubated in low glucose or high glucose medium with or without 100 nM TAK-242 for 18 h and then the healing distance was measured. (E) Enhanced cell migration under the high glucose condition with the TLR4 inhibitor. A total of 3x10^5^ RPTC were seeded in a transwell insert, which was put in a 24-well plate containing 600 μL of medium with or without 100 nM TAK-242 in low glucose or high glucose medium for 6 h. The cells that migrated to the undersurface of the insert were stained with PI and counted. In C, D and E, data are expressed as the mean ± S.D. (n = 4). *, p<0.05 versus the low glucose group; **, p<0.05 versus the high glucose group without TAK-242 treatment.

Based on the above observations, we hypothesized that the upregulation of TLR4 may contribute to defective wound healing in high-glucose-treated cell cultures. To test this possibility, we first examined the effects of a TLR4 inhibitor on wound healing. If high glucose levels suppress wound healing via the upregulation of TLR4, then a TLR4 inhibitor would enhance wound healing under high glucose conditions. We first titrated the concentrations of TAK-242, a selective TLR4 inhibitor [18], and found that 100 nM of TAK-242 at 37°C for 12 h effectively inhibited TLR4 upregulation under high glucose conditions (Fig 5A). MyD88 (Fig 5B) and TLR4 mRNA (Fig 5C) upregulation was also inhibited by TAK-242 under high glucose conditions. We further examined the effects of this TLR4 inhibitor on scratch-wound healing and transwell migration under high glucose conditions. As shown in Fig 5D, TAK-242 significantly promoted wound healing. In addition, the impairment of migration under the high glucose condition was reversed by TAK-242 (Fig 5E), suggesting that TLR4 might contribute to the inhibition of wound healing under high glucose conditions.

### TLR4 knockdown reverses wound healing defects in high-glucose-treated RPTC

**Fig 6:**
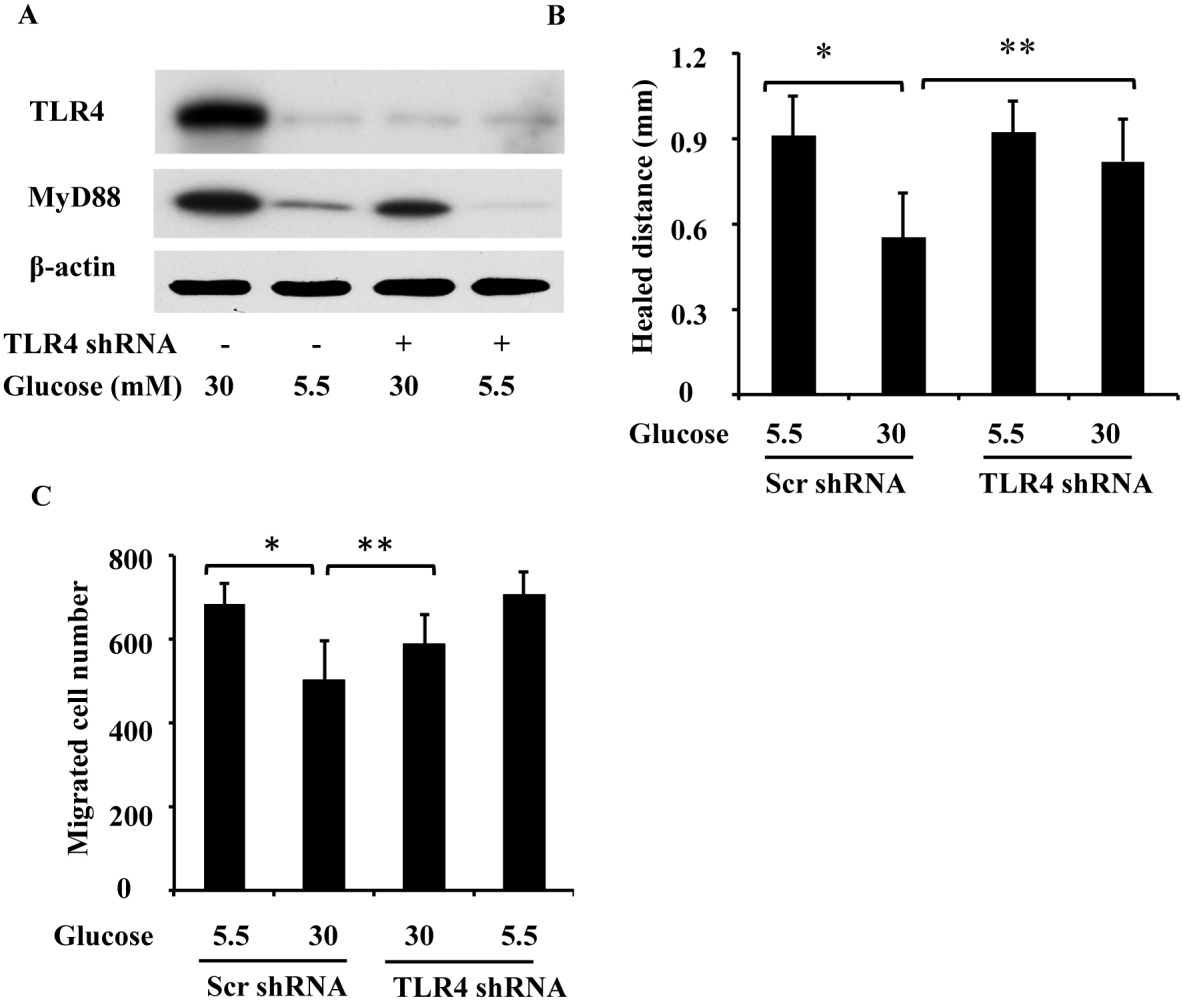
TLR4 shRNA abrogates impaired wound healing in high-glucose-treated RPTC. RPTC were infected with lentiviruses containing a scrambled control sequence (Scr) or the TLR4 shRNA sequence (shRNA) and then cultured for 2 days in low glucose or high glucose medium for the following experiments. (A) TLR4 knockdown caused by the TLR4 shRNA lentivirus. After infection and a high glucose treatment, whole-cell lysates were collected for an immunoblot analysis of TLR4 and MyD88. (B) Scratch-wound healing. After lentiviral infection, RPTC were scratch-wounded and incubated in low glucose or high glucose medium for 18 h to measure the distance over which healing occurred. (C) Transwell cell migration. After lentiviral infection, a total of 3x10^5^ RPTC were seeded in a transwell insert, which was put in a 24-well plate containing 600 μL of low glucose or high glucose medium for 6 h. The cells that migrated to the undersurface were stained with PI and counted. In B and C, data are expressed as the mean ± S.D. (n = 4). *, p<0.05 versus the low glucose group; **, p<0.05 versus high glucose group infected with the scrambled sequence.

To further determine the role of TLR4 in wound healing defects under high glucose conditions, we tested the effect of TLR4 knockdown using shRNA. RPTC were infected with lentiviruses containing TLR4 shRNA or a scrambled control sequence, cultured in low glucose and high glucose medium, and then subjected to scratch wounding and transwell migration. Based on an immunoblot analysis, TLR4 shRNA, but not the scrambled sequence, reduced TLR4 and MyD88 expression in RPTC (Fig 6A). It is noteworthy that although high glucose levels suppressed wound healing in cells transfected with the scrambled sequence, the suppressive effect was reversed by shRNA-mediated TLR4 knockdown (Fig 6B). In addition, the impaired migration under the high glucose conditions was reversed by TLR4 shRNA (Fig 6C). The restoration of wound healing in high-glucose-treated cells by knocking down TLR4 supports a role for TLR4 in wound healing defects under high glucose conditions.

### TLR4 overexpression suppresses wound healing in low-glucose-treated RPTC

**Fig 7:**
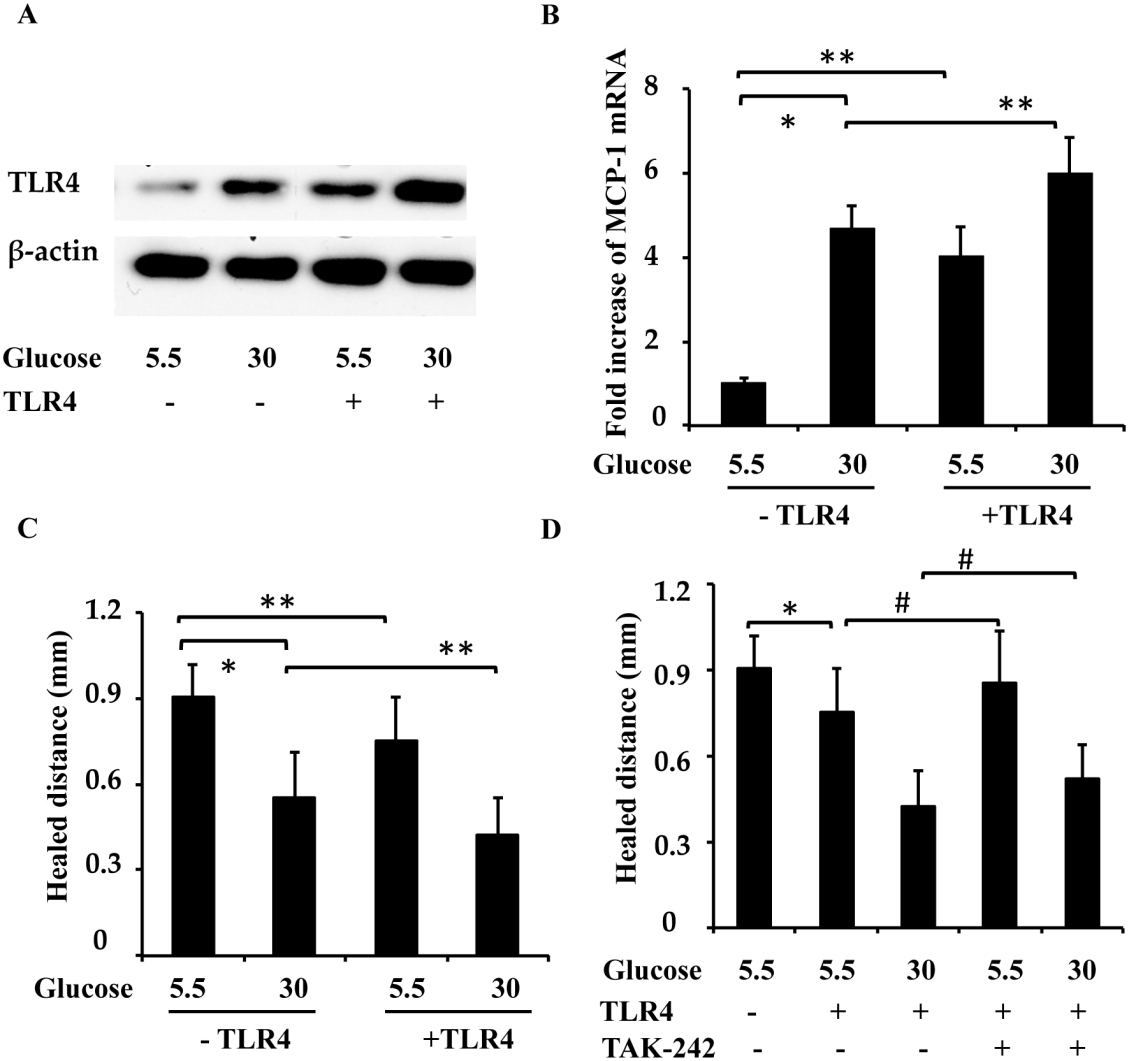
Overexpression of TLR4 in low-glucose-treated RPTC suppresses wound healing. RPTC were infected with lentiviruses containing TLR4 or control lentivirus and then subjected to low glucose and high glucose treatment for 2 days, followed by a scratch-wound healing assay. (A) Increased TLR4 levels were detected in the low-glucose- and high-glucose-treated RPTC. (B) MCP-1 mRNA level in low-glucose-treated and high-glucose-treated RPTC cells after TLR4 overexpression. (C) Overexpression of TLR4 in low-glucose-treated cells also suppressed wound healing, mimicking the effect of high glucose levels. (D) RPTC cells were infected with lentivirus containing TLR4 or control lentivirus, then cultured for 18 h in low glucose or high glucose medium with or without TAK-242, followed by a scratch-wound healing assay. Data are expressed as the mean ± S.D. (n = 4). *, p<0.05 versus the low glucose group; #, p<0.05 versus the group without TAK-242.

The above results (Figs 5 and 6) suggest that TLR4 inhibition promotes wound healing in high-glucose-treated cells. To further examine the role of TLR4 on wound healing, we determined the effect of TLR4 overexpression on wound healing in low-glucose-treated cells. RPTC were infected with lentiviruses containing TLR4, and then subjected to low glucose or high glucose treatments, followed by a scratch-wound healing assay. As shown in Fig 7A, increased TLR4 levels were detected in the low-glucose-treated RPTC, and MCP-1 mRNA levels were also increased in the low-glucose-treated RPTC after TLR4 overexpression, indicating TLR4 signaling activation. Overexpression of TLR4 in low-glucose-treated cells also suppressed wound healing, mimicking the effects of high glucose levels (Fig 7C). TLR4 inhibitor, TAK-242, blocked this effect of overexpression (Fig 7D). However, TLR4 overexpression further inhibited wound healing under the high glucose condition (Fig 7C). These results further confirm the involvement of TLR4 in wound healing defects under high glucose conditions.

## Discussion

Traditionally, chronic kidney disease (CKD) and acute kidney injury (AKI) are classified as two separate kidney diseases. However, recent epidemiological research has revealed a bidirectional relationship between them[19]. CKD patients are predisposed to AKI and AKI leads to a much worse prognosis in CKD patients (including those with diabetes) than prognosis in non-CKD patients[1]. Our recently published research demonstrates that renal ischemia and reperfusion induced more severe AKI in diabetic mice than in nondiabetic mice and that the severity of AKI is correlated with their blood glucose levels[3]. However, the pathophysiology of AKI includes kidney tubular cell death and repair[4], and whether and how kidney repair after AKI is affected by diabetes are not well understood. Therefore, we hypothesized that diabetes inhibits kidney repair after AKI, leading to lower recovery rates and higher mortality rates. Our unpublished studies suggested that TLR4 activation under high glucose conditions contributed to the higher apoptosis induced by ATP-depletion in tubular cells, and TLR4 played important roles in the higher sensitivity of high glucose-conditioned cells to acute injury, but roles of TLR4 in kidney repair under diabetic or high glucose conditions were unclear. In the present study, we investigated the effects of high glucose on wound healing in kidney tubular cells *in vitro*. We first observed that high glucose levels delayed epithelial wound closure (Fig 1) and migration (Fig 2) in three different kidney tubular cell lines (RPTC, NRK-52E, and HEK 293 cells), and induced TLR4 expression in a time-dependent manner. Further study showed that MyD88, p-p38 MAPK, and MCP-1 levels were increased after high glucose treatment, and TLR4 upregulation in response to high glucose levels was through PKC activation (Fig 4). Next, we downregulated TLR4 levels with a pharmacological inhibitor or TLR4 shRNA in RPTC under high glucose conditions. Interestingly, both the TLR4 inhibitor (Fig 5) and shRNA (Fig 6) promoted wound healing under these conditions. In contrast, the overexpression of TLR4 in low-glucose-treated RPTC suppressed wound healing, mimicking the effect of high glucose levels (Fig 7). These results suggest that the upregulation of TLR4 via PKC activation contributes to defective wound healing in high-glucose-treated kidney tubular cells.

In noninfectious inflammatory conditions, TLR4 was activated by interacting with nuclear protein high-mobility group box 1 (HMGB1), which was originally identified as a DNA-binding protein that regulates gene transcription, HMGB1 binding of TLR4 induced NF-κB upregulation [20]. Recently, it has been identified as a TLR4 endogenous ligand in diabetic conditions[21], HMGB1 and TLR4 level was increased in kidney tubular cells in diabetic nephropathy biopsies[15]. HMGB1 was also reported to mediate tubular ischemia-reperfusion injury through TLR4 activation in kidney[22]. In our study, we also detected an increased level of HMGB1 after high glucose treatment in cultured kidney tubular cell lines (S2 Fig), TLR4 might interact with HMGB1 and inhibit kidney repair under high glucose conditions, which needs further investigation.

It is well established that TLR4 plays important and complex roles during acute injury and the following repair processes in many organs. The activation of TLR4 can modify tissue injury and repair in a positive or negative fashion. TLR4 promotes injury in almost all organs, as demonstrated by the protection of TLR4-mutant or TLR4-deficient mice after hepatic, renal, cardiac, and cerebral ischemia-reperfusion[6–8]. However, TLR4 exerts a cytoprotective role and prevents tissue injury in the lungs and intestines under stress conditions[9, 10]. In skin wounds, TLR4 becomes upregulated following injury and slowly decreases to baseline by day 10 and is primarily located in epidermal keratinocytes [11, 12], TLR4-deficient mice show impaired wound healing in skin[11], indicating that TLR4 promotes wound healing in skin. However, under diabetic conditions, TLR4-deficient mice with induced diabetes show significantly improved wound healing compared to diabetic wild-type animals in skin[13], suggesting that TLR4 inhibits skin wound healing under high glucose conditions and that TLR4 contributes to impaired skin wound healing at high glucose levels.

There is accumulating evidence suggesting that TLR4 aggravates renal dysfunction in acute and chronic kidney diseases[6, 23]. TLR4 has been shown to be induced in tubular epithelial cells in the kidney following ischemia in a MyD88-dependent manner, and TLR4-deficient mice are protected against tubular damage[6], suggesting that TLR4 promotes renal ischemia-reperfusion injury[6, 14]. Under high glucose or diabetic conditions, Lin *et al*. demonstrated that high glucose levels induce TLR4 expression in a time- and dose-dependent manner in human proximal tubular epithelial cells *in vitro*, resulting in the upregulation of IL-6 and chemokine (C-C motif) ligand 2 (CCL-2) expression via NF-κB activation, and further study showed that animal and human kidneys with diabetic nephropathy had increased tubular TLR4 expression, while TLR4-deficient mice demonstrated significantly ameliorated albuminuria and renal dysfunction, suggesting that TLR4 activation is involved in diabetic kidney injury. To date, the role of TLR4 in kidney repair in diabetic or high glucose conditions is not well understood. In human monocytes, high glucose induced TLR4 expression via PKC-δ by stimulating NADPH oxidase[24]. In our study, Toll-like receptor 4 (TLR4), MyD88, p-p38 and phospho-protein kinase C (PKC) levels were upregulated after high glucose treatment, and the PKC inhibitor staurosporine suppressed TLR4 and p-p38 upregulation under high glucose conditions, indicating that high glucose levels enhances TLR4 expression via PKC activation.

One of the major symptoms of diabetes mellitus is delayed wound healing. Cell migration and proliferation are important processes during wound healing. High glucose levels inhibit wound healing in many tissues and cells, including peritoneal mesothelial cells[25] and fibroblasts[26]. In skin wounds, high glucose levels inhibit cell migration by delaying migration speed in rat fibroblasts[26], and they inhibit human fibroblast cell migration in wound healing via the repression of bFGF-regulating JNK phosphorylation[27]. In the peritoneum, high glucose concentrations inhibit the FAK-mediated migration of mesothelial cells[25]. In corneal cells, high glucose levels impair the EGFR—phosphatidylinositol 3-kinase/Akt pathway through ROS-induced effects, resulting in delayed corneal epithelial wound healing[28]. However, how high glucose levels affect kidney repair is unknown. Using scratch-wound healing and transwell models, we demonstrated that high glucose levels significantly delay scratch-wound healing and transwell migration in cultured kidney tubular cells.

The role of TLR4 in wound healing is very complex and varies between cell and tissue types, with many studies suggesting that TLR4 is an important regulator of inflammatory signals in wound healing in different tissues and cells[5]. In the liver, MyD88 is required for the original liver mass restoration after partial hepatectomy, but TLR4 is not involved in liver regeneration[29]. On the other hand, TLR4 activation in response to high LPS doses inhibits liver regeneration, suggesting that TLR signaling can modulate liver regeneration in positive and negtive directions depending on the degree of TLR activation[30]. In the intestine, TLR4/MyD88 signaling is required for epithelial regeneration following DSS-induced injury[9]. In excisional skin wounds, MyD88-deficient mice show markedly slower healing rates than wild-type mice, suggesting that MyD88 promotes wound healing[31]. Moreover, a number of studies support a role for TLR4 in promoting fibrogenic responses in vivo and in vitro, TLR4-deficient mice have been shown to have significantly decreased hepatic fibrogenesis induced by bile duct ligation[32]. The role of TLR4 on kidney repair after injury remains unknown. Our research is the first to show that high glucose levels delay scratch wound healing and transwell migration in cultured renal tubular cells and that this is accompanied by TLR4 upregulation. In addition, we show that the pharmacological inhibition of TLR4 or shRNA-mediated TLR4 knockdown improves wound healing and transwell migration under high glucose conditions. The overexpression of TLR4 in low-glucose-treated cells also suppresses wound healing, mimicking the effect of high glucose levels.

We demonstrated that TLR4 contributes to delayed wound healing in renal proximal tubular cells under high glucose conditions. However, the signaling cascades mediating the inhibitory effects of high glucose levels on tubular cell repair have not been clearly characterized. In the intestine, TLR4/MyD88 signaling is required for the regeneration of epithelia following DSS-induced injury[9]. This signaling is transmitted through cyclooxygenase 2 (Cox-2)-expressing macrophages that migrate to stimulate epithelial progenitor cell proliferation[33]. After DSS injury, Cox-2 expression is increased in wild-type mice, but not in TLR4-deficient mice[9]. Moreover, prostaglandin E2 (PGE2) is a key product of Cox-2, and supplementation of TLR4-deficient mice with PGE2 completely restores defective proliferation[9]. Accordingly, the Cox-2 inhibitor decreased LPS-induced proliferation in intestinal cells[9]. In the liver, a deficiency of MyD88 delays the expression of growth-promoting genes, such as c-fos, c-jun, JunB and c-myc, and suppresses the activation of NF-**κ**B[29, 34]. In the early stages of a skin wound, the injury induces TLR4 expression and the phosphorylation of p38, JNK, and MAPK, and the blockade of TLR4 delays keratinocytes migration and abolishes the phosphorylation of p38, JNK, and MAPK[11]. In our study, high glucose induces TLR4, MyD88 expression, p38 phosphorylation, and target gene MCP-1 mRNA expression, inhibition of TLR4 delayed promotes wound healing and abolishes the phosphorylation of p38 under high glucose conditions.

In this study, we tested the hypothesis that high glucose levels impair wound healing in the kidney after AKI and found that scratch-wound healing and transwell migrations are impaired under high glucose conditions, which is accompanied by the upregulation of TLR4, MyD88 and p-p38 MAPK via PKC activation. In addition, the inhibition of TLR4 with a pharmacological inhibitor or shRNA promotes wound healing under high glucose conditions, and the overexpression of TLR4 in low-glucose-treated cells also suppresses wound healing, mimicking the effect of high glucose levels, suggesting that TLR4 contributes to the delayed wound healing of renal proximal tubular cells under high glucose conditions *in vitro*.

## Supporting information

S1 Fig Scratch-wound healing at different time-points in cultured kidney tubular cells. A monolayer of confluent RPTC grown in a 35-mm dish was linearly scratched with a sterile 1000 μL pipette tip. Phase-contrast images were recorded at 0h, 2.5h, 5h and 10h after scratching. Results showed that there was no obvious proliferation at first 6 hours.
(PPTX)

S2 Fig HMGB1 upregulation during high glucose treatment in RPTC. HMGB1 mRNA levels were examined via real-time PCR after high glucose treatment at different time-points. HMGB1 mRNA upregulation in the high glucose medium. Data are expressed as the mean ± S.D. (n≥3). *, p<0.05 versus the control (0 h).
(PPTX)

## References

[1] Thakar CV, Christianson A, Himmelfarb J, Leonard AC. Acute kidney injury episodes and chronic kidney disease risk in diabetes mellitus. Clinical journal of the American Society of Nephrology: CJASN. 2011;6(11):2567–72. Epub 2011/09/10. 10.2215/CJN.01120211 ; 21903988 PMC3359576

[2] Goor Y, Peer G, Iaina A, Blum M, Wollman Y, Chernihovsky T, et al Nitric oxide in ischaemic acute renal failure of streptozotocin diabetic rats. Diabetologia. 1996;39(9):1036–40. Epub 1996/09/01. . 8877286 10.1007/BF00400651

[3] Peng J, Li X, Zhang D, Chen JK, Su Y, Smith SB, et al Hyperglycemia, p53, and mitochondrial pathway of apoptosis are involved in the susceptibility of diabetic models to ischemic acute kidney injury. Kidney international. 2015;87(1):137–50. Epub 2014/06/26. 10.1038/ki.2014.226 ; 24963915 PMC4276728

[4] Bonventre JV. Pathophysiology of AKI: injury and normal and abnormal repair. Contributions to nephrology. 2010;165:9–17. Epub 2010/04/30. 10.1159/000313738 . 20427950

[5] Kluwe J, Mencin A, Schwabe RF. Toll-like receptors, wound healing, and carcinogenesis. J Mol Med (Berl). 2009;87(2):125–38. Epub 2008/12/18. 10.1007/s00109-008-0426-z ; 19089397 PMC2791674

[6] Wu H, Chen G, Wyburn KR, Yin J, Bertolino P, Eris JM, et al TLR4 activation mediates kidney ischemia/reperfusion injury. The Journal of clinical investigation. 2007;117(10):2847–59. Epub 2007/09/15. 10.1172/JCI31008 ; 17853945 PMC1974864

[7] Tang SC, Arumugam TV, Xu X, Cheng A, Mughal MR, Jo DG, et al Pivotal role for neuronal Toll-like receptors in ischemic brain injury and functional deficits. Proceedings of the National Academy of Sciences of the United States of America. 2007;104(34):13798–803. Epub 2007/08/19. 10.1073/pnas.0702553104 ; 17693552 PMC1959462

[8] Oyama J, Blais C Jr., Liu X, Pu M, Kobzik L, Kelly RA, et al Reduced myocardial ischemia-reperfusion injury in toll-like receptor 4-deficient mice. Circulation. 2004;109(6):784–9. Epub 2004/02/19. 10.1161/01.CIR.0000112575.66565.84 . 14970116

[9] Fukata M, Chen A, Klepper A, Krishnareddy S, Vamadevan AS, Thomas LS, et al Cox-2 is regulated by Toll-like receptor-4 (TLR4) signaling: Role in proliferation and apoptosis in the intestine. Gastroenterology. 2006;131(3):862–77. Epub 2006/09/06. 10.1053/j.gastro.2006.06.017 ; 16952555 PMC2169292

[10] Jiang D, Liang J, Fan J, Yu S, Chen S, Luo Y, et al Regulation of lung injury and repair by Toll-like receptors and hyaluronan. Nature medicine. 2005;11(11):1173–9. Epub 2005/10/26. 10.1038/nm1315 . 16244651

[11] Chen L, Guo S, Ranzer MJ, DiPietro LA. Toll-like receptor 4 has an essential role in early skin wound healing. The Journal of investigative dermatology. 2013;133(1):258–67. Epub 2012/09/07. 10.1038/jid.2012.267 ; 22951730 PMC3519973

[12] Dasu MR, Isseroff RR. Toll-like receptors in wound healing: location, accessibility, and timing. The Journal of investigative dermatology. 2012;132(8):1955–8. Epub 2012/07/17. 10.1038/jid.2012.208 . 22797299

[13] Dasu MR, Jialal I. Amelioration in wound healing in diabetic toll-like receptor-4 knockout mice. Journal of diabetes and its complications. 2013;27(5):417–21. Epub 2013/06/19. 10.1016/j.jdiacomp.2013.05.002 ; 23773694 PMC3770740

[14] Chen J, John R, Richardson JA, Shelton JM, Zhou XJ, Wang Y, et al Toll-like receptor 4 regulates early endothelial activation during ischemic acute kidney injury. Kidney international. 2011;79(3):288–99. Epub 2010/10/12. 10.1038/ki.2010.381 ; 20927041 PMC3404515

[15] Lin M, Yiu WH, Wu HJ, Chan LY, Leung JC, Au WS, et al Toll-like receptor 4 promotes tubular inflammation in diabetic nephropathy. Journal of the American Society of Nephrology: JASN. 2012;23(1):86–102. Epub 2011/10/25. 10.1681/ASN.2010111210 ; 22021706 PMC3269929

[16] Sun L, Xie P, Wada J, Kashihara N, Liu FY, Zhao Y, et al Rap1b GTPase ameliorates glucose-induced mitochondrial dysfunction. Journal of the American Society of Nephrology: JASN. 2008;19(12):2293–301. Epub 2008/08/30. 10.1681/ASN.2008030336 ; 18753253 PMC2588105

[17] Peng J, Ramesh G, Sun L, Dong Z. Impaired wound healing in hypoxic renal tubular cells: roles of hypoxia-inducible factor-1 and glycogen synthase kinase 3beta/beta-catenin signaling. The Journal of pharmacology and experimental therapeutics. 2012;340(1):176–84. Epub 2011/10/20. 10.1124/jpet.111.187427 ; 22010210 PMC3251027

[18] Matsunaga N, Tsuchimori N, Matsumoto T, Ii M. TAK-242 (resatorvid), a small-molecule inhibitor of Toll-like receptor (TLR) 4 signaling, binds selectively to TLR4 and interferes with interactions between TLR4 and its adaptor molecules. Molecular pharmacology. 2011;79(1):34–41. Epub 2010/10/01. 10.1124/mol.110.068064 . 20881006

[19] Chawla LS, Kimmel PL. Acute kidney injury and chronic kidney disease: an integrated clinical syndrome. Kidney international. 2012;82(5):516–24. Epub 2012/06/08. 10.1038/ki.2012.208 . 22673882

[20] Klune JR, Dhupar R, Cardinal J, Billiar TR, Tsung A. HMGB1: endogenous danger signaling. Mol Med. 2008;14(7–8):476–84. Epub 2008/04/24. 10.2119/2008-00034.Klune ; 18431461 PMC2323334

[21] Dasu MR, Devaraj S, Park S, Jialal I. Increased toll-like receptor (TLR) activation and TLR ligands in recently diagnosed type 2 diabetic subjects. Diabetes care. 2010;33(4):861–8. Epub 2010/01/14. 10.2337/dc09-1799 ; 20067962 PMC2845042

[22] Wu H, Ma J, Wang P, Corpuz TM, Panchapakesan U, Wyburn KR, et al HMGB1 contributes to kidney ischemia reperfusion injury. Journal of the American Society of Nephrology: JASN. 2010;21(11):1878–90. Epub 2010/09/18. 10.1681/ASN.2009101048 ; 20847143 PMC3014003

[23] Cunningham PN, Wang Y, Guo R, He G, Quigg RJ. Role of Toll-like receptor 4 in endotoxin-induced acute renal failure. J Immunol. 2004;172(4):2629–35. Epub 2004/02/07. . 14764737 10.4049/jimmunol.172.4.2629

[24] Dasu MR, Devaraj S, Zhao L, Hwang DH, Jialal I. High glucose induces toll-like receptor expression in human monocytes: mechanism of activation. Diabetes. 2008;57(11):3090–8. Epub 2008/07/25. 10.2337/db08-0564 ; 18650365 PMC2570406

[25] Tamura M, Osajima A, Nakayamada S, Anai H, Kabashima N, Kanegae K, et al High glucose levels inhibit focal adhesion kinase-mediated wound healing of rat peritoneal mesothelial cells. Kidney international. 2003;63(2):722–31. Epub 2003/03/13. 10.1046/j.1523-1755.2003.00772.x . 12631140

[26] Lamers ML, Almeida ME, Vicente-Manzanares M, Horwitz AF, Santos MF. High glucose-mediated oxidative stress impairs cell migration. PloS one. 2011;6(8):e22865 Epub 2011/08/10. 10.1371/journal.pone.0022865 ; 21826213 PMC3149607

[27] Xuan YH, Huang BB, Tian HS, Chi LS, Duan YM, Wang X, et al High-glucose inhibits human fibroblast cell migration in wound healing via repression of bFGF-regulating JNK phosphorylation. PloS one. 2014;9(9):e108182 Epub 2014/09/23. 10.1371/journal.pone.0108182 ; 25244316 PMC4171528

[28] Xu KP, Li Y, Ljubimov AV, Yu FS. High glucose suppresses epidermal growth factor receptor/phosphatidylinositol 3-kinase/Akt signaling pathway and attenuates corneal epithelial wound healing. Diabetes. 2009;58(5):1077–85. Epub 2009/02/04. 10.2337/db08-0997 ; 19188434 PMC2671049

[29] Seki E, Tsutsui H, Iimuro Y, Naka T, Son G, Akira S, et al Contribution of Toll-like receptor/myeloid differentiation factor 88 signaling to murine liver regeneration. Hepatology. 2005;41(3):443–50. Epub 2005/02/22. 10.1002/hep.20603 . 15723296

[30] Sun R, Gao B. Negative regulation of liver regeneration by innate immunity (natural killer cells/interferon-gamma). Gastroenterology. 2004;127(5):1525–39. Epub 2004/11/03. . 15521020 10.1053/j.gastro.2004.08.055

[31] Macedo L, Pinhal-Enfield G, Alshits V, Elson G, Cronstein BN, Leibovich SJ. Wound healing is impaired in MyD88-deficient mice: a role for MyD88 in the regulation of wound healing by adenosine A2A receptors. The American journal of pathology. 2007;171(6):1774–88. Epub 2007/11/03. 10.2353/ajpath.2007.061048 ; 17974599 PMC2111102

[32] Seki E, De Minicis S, Osterreicher CH, Kluwe J, Osawa Y, Brenner DA, et al TLR4 enhances TGF-beta signaling and hepatic fibrosis. Nature medicine. 2007;13(11):1324–32. Epub 2007/10/24. 10.1038/nm1663 . 17952090

[33] Brown SL, Riehl TE, Walker MR, Geske MJ, Doherty JM, Stenson WF, et al Myd88-dependent positioning of Ptgs2-expressing stromal cells maintains colonic epithelial proliferation during injury. The Journal of clinical investigation. 2007;117(1):258–69. Epub 2007/01/04. 10.1172/JCI29159 ; 17200722 PMC1716207

[34] Campbell JS, Riehle KJ, Brooling JT, Bauer RL, Mitchell C, Fausto N. Proinflammatory cytokine production in liver regeneration is Myd88-dependent, but independent of Cd14, Tlr2, and Tlr4. J Immunol. 2006;176(4):2522–8. Epub 2006/02/04. . 16456013 10.4049/jimmunol.176.4.2522

