# Supplementary material for: Upregulation of TLR4 via PKC activation contributes to impaired wound healing in high-glucose-treated kidney proximal tubular cells
Source: PLoS One. 2017 May 24;12(5):e0178147. doi: 10.1371/journal.pone.0178147 (PMC5443579; doi:10.1371/journal.pone.0178147)
Supplement: S1 Fig — A monolayer of confluent RPTC grown in a 35-mm dish was linearly scratched with a sterile 1000 μL pipette tip. Phase-contrast images were recorded at 0h, 2.5h, 5h and 10h after scratching. Results showed that there was no obvious proliferation at first 6 hours. (PPTX) [file pone.0178147.s001.pptx]

## Slide 1
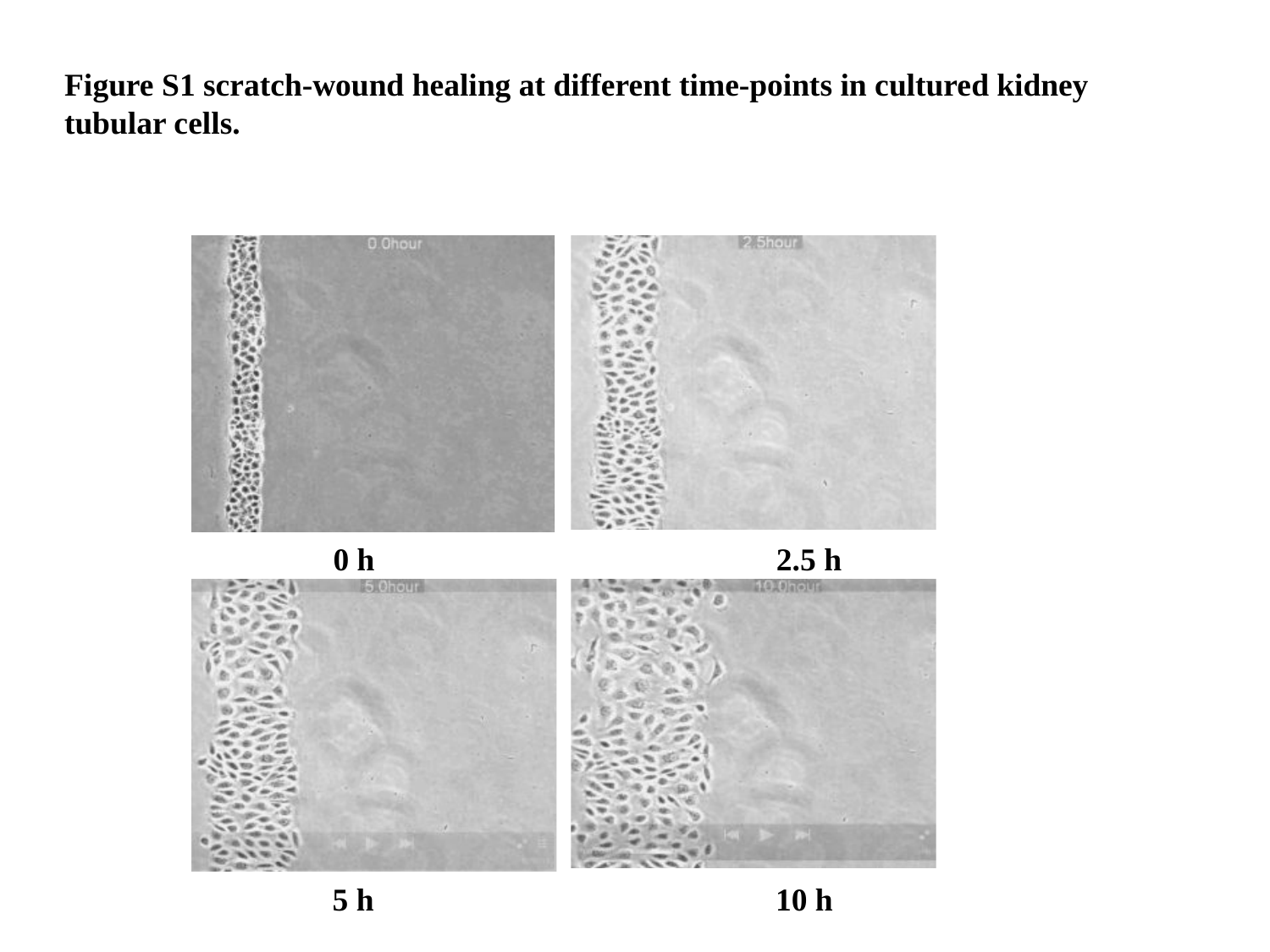

Figure S1 scratch-wound healing at different time-points in cultured kidney tubular cells.
0 h 2.5 h
5 h 10 h
