# Supplementary material for: Upregulation of TLR4 via PKC activation contributes to impaired wound healing in high-glucose-treated kidney proximal tubular cells
Source: PLoS One. 2017 May 24;12(5):e0178147. doi: 10.1371/journal.pone.0178147 (PMC5443579; doi:10.1371/journal.pone.0178147)
Supplement: S2 Fig — HMGB1 mRNA levels were examined via real-time PCR after high glucose treatment at different time-points. HMGB1 mRNA upregulation in the high glucose medium. Data are expressed as the mean ± S.D. (n≥3). *, p<0.05 versus the control (0 h). (PPTX) [file pone.0178147.s002.pptx]

## Slide 1
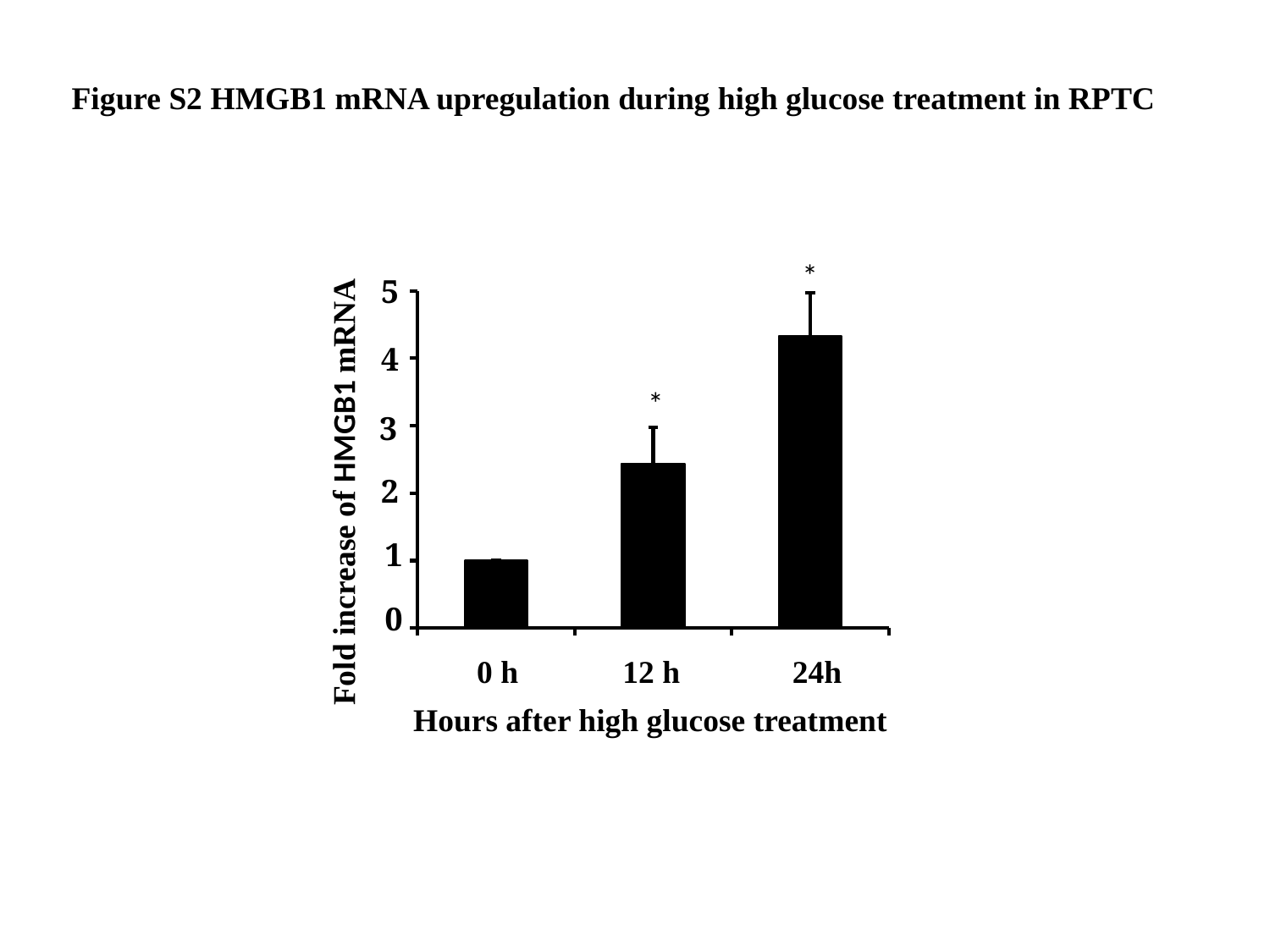

Figure S2 HMGB1 mRNA upregulation during high glucose treatment in RPTC
### Chart
| Category | HMGB1 mRNA |
|---|---|
| 30mM 0h | 1.0 |
| 30mM 12h | 2.433232 |*
5
4
*
3
2
Fold increase of HMGB1 mRNA
1
0
0 h 12 h 24h
Hours after high glucose treatment
